# Bis{6,6′-dimeth­oxy-2,2′-[ethane-1,2-diyl­bis(imino­methyl­ene)]diphenolato(1.5−)-κ^4^ *O*,*N*,*N*′,*O*′}erbium(III)

**DOI:** 10.1107/S1600536809001421

**Published:** 2009-01-17

**Authors:** Hai-Tao Xia, De-Fu Rong, Ying-Ying Zhang, Shu-Ping Yang, Da-Qi Wang

**Affiliations:** a School of Chemical Engineering, Huaihai Institute of Technology, Lianyungang 222005, People’s Republic of China; b Beilun Entry–Exit Inspection and Quarantine Bureau of China, Ningbo Zhejiang, People’s Republic of China; c College of Chemistry and Chemical Engineering, Liaocheng University, Shandong 252059, People’s Republic of China

## Abstract

In the title compound, [Er(C_18_H_22.5_N_2_O_4_)_2_], the Er atom is located on a twofold rotation axis and is eight-coordinated by four O atoms and four N atoms from two symmetry-related 6,6′-dimethoxy-2,2′-(ethane-1,2-diyldiiminodimethylene)diphenolate(1.5−) ligands. Due to disorder of one phenolate H atom with half-occupation, the overall charge of one tetradentate ligand is −1.5. The ligand molecules are stabilised by intramolecular N—H⋯O and O—H⋯O hydrogen bonds and are linked into a chain parallel to the *a* axis by a C—H⋯O hydrogen bond. Neighbouring chains are connected by van der Waals forces, resulting in a three-dimensional network.

## Related literature

For related structures, see: Liu *et al.* (2007 ▶); Xia *et al.* (2006 ▶). For isotypic structures, see: Xia *et al.* (2009*a* ▶, 2009*b* ▶). 
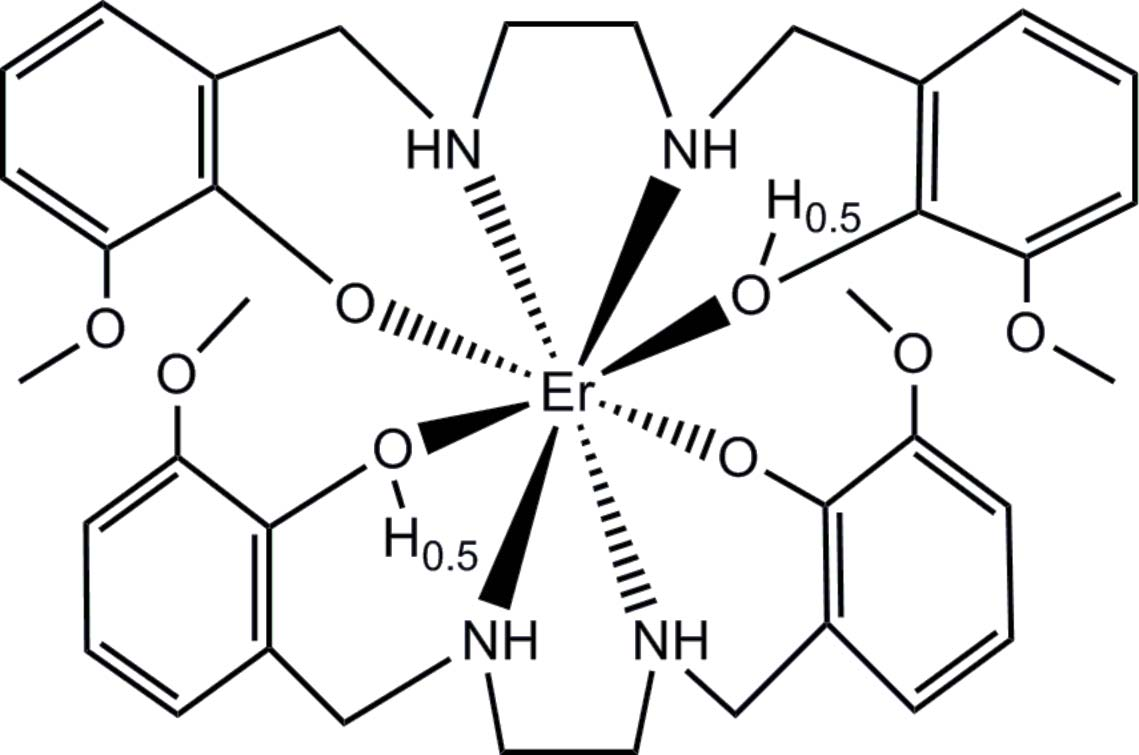


## Experimental

### 

#### Crystal data

[Er(C_18_H_22.5_N_2_O_4_)_2_]
*M* *_r_* = 829.02
Orthorhombic, 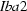

*a* = 11.1542 (10) Å
*b* = 21.958 (2) Å
*c* = 14.1751 (15) Å
*V* = 3471.8 (6) Å^3^
*Z* = 4
Mo *K*α radiation
μ = 2.48 mm^−1^
*T* = 298 (2) K
0.20 × 0.15 × 0.14 mm

#### Data collection

Siemens SMART 1000 CCD area-detector diffractometer
Absorption correction: multi-scan (*SADABS*; Sheldrick, 1996 ▶) *T*_min_ = 0.637, *T*_max_ = 0.723
7986 measured reflections
3007 independent reflections
2199 reflections with *I* > 2σ(*I*)
*R*_int_ = 0.031

#### Refinement

*R*[*F* ^2^ > 2σ(*F* ^2^)] = 0.044
*wR*(*F* ^2^) = 0.162
*S* = 1.06
3007 reflections
224 parameters
1 restraint
H-atom parameters constrained
Δρ_max_ = 0.75 e Å^−3^
Δρ_min_ = −1.42 e Å^−3^
Absolute structure: Flack (1983 ▶), 1400 Friedel pairs
Flack parameter: 0.02 (5)

### 

Data collection: *SMART* (Siemens, 1996 ▶); cell refinement: *SAINT* (Siemens, 1996 ▶); data reduction: *SAINT*; program(s) used to solve structure: *SHELXS97* (Sheldrick, 2008 ▶); program(s) used to refine structure: *SHELXL97* (Sheldrick, 2008 ▶); molecular graphics: *SHELXTL* (Sheldrick, 2008 ▶); software used to prepare material for publication: *SHELXTL*.

## Supplementary Material

Crystal structure: contains datablocks I, global. DOI: 10.1107/S1600536809001421/at2680sup1.cif

Structure factors: contains datablocks I. DOI: 10.1107/S1600536809001421/at2680Isup2.hkl

Additional supplementary materials:  crystallographic information; 3D view; checkCIF report

## Figures and Tables

**Table 1:** Selected bond lengths (Å)

Er1—O3^i^	2.202 (6)
Er1—O3	2.202 (6)
Er1—O1	2.203 (6)
Er1—O1^i^	2.203 (6)
Er1—N2	2.595 (8)
Er1—N2^i^	2.595 (8)
Er1—N1^i^	2.628 (8)
Er1—N1	2.628 (8)

Symmetry code: (i) 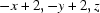.

**Table 2:** Hydrogen-bond geometry (Å, °)

*D*—H⋯*A*	*D*—H	H⋯*A*	*D*⋯*A*	*D*—H⋯*A*
C16—H16*A*⋯O4^ii^	0.96	2.70	3.49 (3)	140
N2—H2⋯O2^i^	0.91	2.34	3.230 (10)	166
N1—H1*C*⋯O4^i^	0.91	2.59	3.462 (12)	162
O1—H1⋯O2	0.82	2.21	2.646 (9)	113

Symmetry codes: (i) 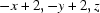; (ii) 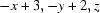.

**Fig. 1.**
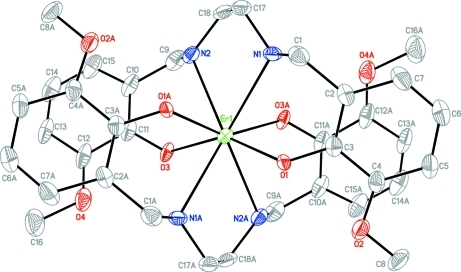
The molecular structure of (I), showing the atom-labelling scheme. Displacement ellipsoids are at the 30% probability level. For clarity, H atoms have been omitted. [symmetry codes: (A) 2 - x, 2 - y, z].

**Fig. 2.**
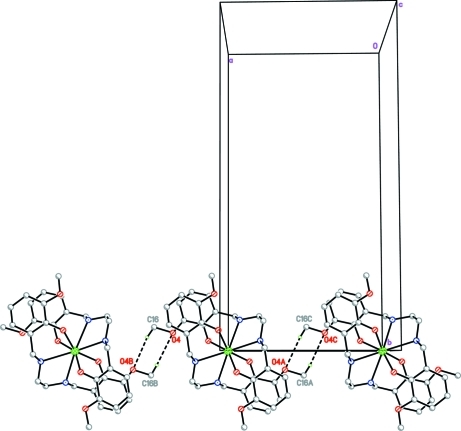
A larger portion of the crystal structure of (I), showing the formation of a hydrogen-bonded chain built from C—H···O. For clarity, H atoms not involved in the hydrogen bonding have been omitted. Dashed lines indicate hydrogen bonds [symmetry codes: (A) 2 - x, 2 - y, z; (B) 3 - x, 2 - y, 2, z; (C) -1 + x, y, z].

## supplementary crystallographic information

### Comment

Diamine derivatives are potentially multidentate ligands. We have recently reported the crystal structure (C_18_H_24_O_2_N_4_) (II) (Xia *et al.*, 2006) which is the ligand of the title compound and a complex [Ce(C_18_H_22_N_2_O_4_)_2_] (III) (Liu *et al.*, 2007). We report here the crystal structure of new rare earth complex (I).

In the title complex (I), the coordination environment of the Er atom and coordination modes of (I) ligands to Er^III^ ion is in agreement with the complexes reported above (Fig. 1). The average bond lengths of between the Erbium center oxygen atoms are 2.203 (6)Å and nitrogen atom are 2.612 (8) Å, longer than the 2.199 (4)Å and shorter than the 2.624 (4)Å of complexes (III), respectively. The dihedral angles between phenyl ring (C2-C7 ring) and anotother phenyl ring are 42.20 (28)°(C10—C15 ring), 42.29 (26)°(C2A—C7A ring) and 15.47 (43)°(C10A—C15A ring) [symmetry codes: (A) 2 - *x*, 2 - *y*, *z*].

In (I), the Er atom is eight-coordinated by four O atoms and four N atoms from two 6,6'-dimethoxy-2,2'-(ethane-1,2-diyldiiminodimethylene)diphenol. The molecules are linked into a chain parallel to the *a* axis by one C—H···O hydrogen bond. Neighbouring chains are connected by van der Waals forces, resulting in a three-dimensional network.

### Experimental

A solution of 6,6'-dimethoxy-2,2'-(ethane-1,2-diyldiiminodimethylene) diphenol (0.328 g, 2 mmol) in ethanol (20 ml), and then a solution of Er(NO_3_)_3_.6H_2_O (0.461 g, 1 mmol) in ethanol (10 ml) was added. The reaction mixture was stirred for 3 h in the air and then filtered. X-ray quality crystals of (I) were obtained by evaporation of an ethanol solution.

### Refinement

The space group was uniquely assigned from the systematic absences. All H atoms were located in difference Fourier maps. H atoms bonded to C, O and N atoms were treated as riding atoms, with C—H distances of 0.93 Å (aryl), 0.96 Å (methyl), 0.97Å (methylene) and N—H distances of 0.90 Å (amino), *U*_iso_(H) = 1.2*U*_eq_(aryl, methylene, NH) or 1.5*U*_eq_(C) (methyl or OH). The H1 bonded to O1 is disordered and were refined with the occupancies ties to 0.5.

### Crystal data

**Table:**

[Er(C_18_H_22.5_N_2_O_4_)_2_]	*F*(000) = 1684
*M**_r_* = 829.02	*D*_x_ = 1.586 Mg m^−^^3^
Orthorhombic, *I**b**a*2	Mo *K*α radiation, λ = 0.71073 Å
Hall symbol: I 2 -2c	Cell parameters from 4129 reflections
*a* = 11.1542 (10) Å	θ = 2.9–25.7°
*b* = 21.958 (2) Å	µ = 2.48 mm^−^^1^
*c* = 14.1751 (15) Å	*T* = 298 K
*V* = 3471.8 (6) Å^3^	Block, brown
*Z* = 4	0.20 × 0.15 × 0.14 mm

### Data collection

**Table:**

Siemens SMART 1000 CCD area-detector diffractometer	3007 independent reflections
Radiation source: fine-focus sealed tube	2199 reflections with *I* > 2σ(*I*)
graphite	*R*_int_ = 0.031
φ and ω scans	θ_max_ = 25.0°, θ_min_ = 1.9°
Absorption correction: multi-scan (*SADABS*; Sheldrick, 1996)	*h* = −13→10
*T*_min_ = 0.637, *T*_max_ = 0.723	*k* = −20→26
7986 measured reflections	*l* = −16→16

### Refinement

**Table:**

Refinement on *F*^2^	Secondary atom site location: difference Fourier map
Least-squares matrix: full	Hydrogen site location: inferred from neighbouring sites
*R*[*F*^2^ > 2σ(*F*^2^)] = 0.044	H-atom parameters constrained
*wR*(*F*^2^) = 0.162	*w* = 1/[σ^2^(*F*_o_^2^) + (0.1143*P*)^2^ + 1.9553*P*] where *P* = (*F*_o_^2^ + 2*F*_c_^2^)/3
*S* = 1.06	(Δ/σ)_max_ = 0.001
3007 reflections	Δρ_max_ = 0.75 e Å^−^^3^
224 parameters	Δρ_min_ = −1.42 e Å^−^^3^
1 restraint	Absolute structure: Flack (1983), 1400 Freidel pairs
Primary atom site location: structure-invariant direct methods	Flack parameter: 0.02 (5)

### Special details

**Table:**

Geometry. All e.s.d.'s (except the e.s.d. in the dihedral angle between two l.s. planes) are estimated using the full covariance matrix. The cell e.s.d.'s are taken into account individually in the estimation of e.s.d.'s in distances, angles and torsion angles; correlations between e.s.d.'s in cell parameters are only used when they are defined by crystal symmetry. An approximate (isotropic) treatment of cell e.s.d.'s is used for estimating e.s.d.'s involving l.s. planes.
Refinement. Refinement of *F*^2^ against ALL reflections. The weighted *R*-factor *wR* and goodness of fit *S* are based on *F*^2^, conventional *R*-factors *R* are based on *F*, with *F* set to zero for negative *F*^2^. The threshold expression of *F*^2^ > σ(*F*^2^) is used only for calculating *R*-factors(gt) *etc*. and is not relevant to the choice of reflections for refinement. *R*-factors based on *F*^2^ are statistically about twice as large as those based on *F*, and *R*- factors based on ALL data will be even larger.

### Fractional atomic coordinates and isotropic or equivalent isotropic displacement parameters (Å^2^)

**Table:**

	*x*	*y*	*z*	*U*_iso_*/*U*_eq_	Occ. (<1)
Er1	1.0000	1.0000	0.0158 (2)	0.0342 (2)	
O1	0.9418 (5)	1.0681 (3)	0.1202 (4)	0.0297 (13)	
H1	0.9919	1.0955	0.1209	0.045*	0.50
O2	0.9141 (6)	1.1865 (3)	0.0933 (5)	0.0479 (18)	
O3	1.1356 (6)	0.9653 (3)	−0.0834 (5)	0.0389 (16)	
O4	1.3687 (7)	0.9386 (4)	−0.0660 (7)	0.065 (3)	
N1	0.7906 (7)	0.9657 (4)	0.0823 (6)	0.040 (2)	
H1C	0.7358	0.9838	0.0439	0.048*	
N2	0.9283 (8)	0.8957 (4)	−0.0492 (6)	0.043 (2)	
H2	0.9673	0.8670	−0.0146	0.052*	
C1	0.7624 (12)	0.9901 (6)	0.1789 (10)	0.049 (3)	
H1A	0.6906	0.9706	0.2029	0.058*	
H1B	0.8279	0.9809	0.2216	0.058*	
C2	0.7440 (9)	1.0568 (5)	0.1748 (7)	0.042 (3)	
C3	0.8366 (9)	1.0926 (5)	0.1404 (7)	0.039 (3)	
C4	0.8159 (9)	1.1552 (5)	0.1290 (7)	0.041 (2)	
C5	0.7089 (10)	1.1808 (6)	0.1508 (8)	0.051 (3)	
H5	0.6957	1.2223	0.1429	0.061*	
C6	0.6169 (10)	1.1415 (7)	0.1866 (9)	0.056 (3)	
H6	0.5428	1.1582	0.2021	0.067*	
C7	0.6330 (9)	1.0854 (7)	0.1977 (9)	0.057 (3)	
H7	0.5708	1.0618	0.2216	0.068*	
C8	0.8935 (13)	1.2474 (5)	0.0637 (10)	0.064 (3)	
H8A	0.8112	1.2519	0.0451	0.095*	
H8B	0.9447	1.2567	0.0112	0.095*	
H8C	0.9107	1.2747	0.1149	0.095*	
C9	0.9606 (16)	0.8829 (7)	−0.1469 (9)	0.061 (4)	
H9A	0.9455	0.9184	−0.1860	0.073*	
H9B	0.9135	0.8492	−0.1709	0.073*	
C10	1.0949 (14)	0.8668 (6)	−0.1482 (8)	0.054 (3)	
C11	1.1731 (11)	0.9109 (5)	−0.1134 (7)	0.043 (3)	
C12	1.2992 (13)	0.8958 (6)	−0.1052 (8)	0.056 (3)	
C13	1.3345 (14)	0.8375 (6)	−0.1369 (9)	0.063 (4)	
H13	1.4149	0.8264	−0.1333	0.076*	
C14	1.2544 (16)	0.7976 (7)	−0.1721 (10)	0.070 (4)	
H14	1.2804	0.7590	−0.1897	0.084*	
C15	1.1369 (14)	0.8120 (6)	−0.1826 (9)	0.066 (4)	
H15	1.0850	0.7852	−0.2128	0.079*	
C16	1.4901 (11)	0.9202 (11)	−0.0443 (19)	0.092 (7)	
H16A	1.5326	0.9538	−0.0167	0.139*	
H16B	1.5298	0.9078	−0.1013	0.139*	
H16C	1.4885	0.8868	−0.0007	0.139*	
C17	0.7704 (9)	0.9017 (5)	0.0703 (9)	0.051 (3)	
H17A	0.6876	0.8920	0.0849	0.061*	
H17B	0.8216	0.8789	0.1127	0.061*	
C18	0.7979 (11)	0.8845 (5)	−0.0316 (10)	0.052 (3)	
H18A	0.7792	0.8419	−0.0422	0.063*	
H18B	0.7498	0.9089	−0.0743	0.063*	

### Atomic displacement parameters (Å^2^)

**Table:**

	*U*^11^	*U*^22^	*U*^33^	*U*^12^	*U*^13^	*U*^23^
Er1	0.0382 (3)	0.0267 (3)	0.0377 (3)	0.0000 (2)	0.000	0.000
O1	0.018 (3)	0.033 (4)	0.038 (3)	0.003 (3)	0.005 (3)	−0.004 (3)
O2	0.053 (4)	0.035 (4)	0.055 (4)	0.009 (3)	0.001 (3)	−0.007 (3)
O3	0.056 (4)	0.026 (4)	0.034 (4)	0.014 (3)	0.011 (3)	−0.001 (3)
O4	0.055 (5)	0.055 (6)	0.085 (7)	0.019 (4)	0.031 (4)	0.023 (5)
N1	0.031 (4)	0.038 (5)	0.052 (5)	−0.009 (4)	−0.007 (4)	0.012 (4)
N2	0.051 (6)	0.026 (4)	0.052 (5)	−0.003 (4)	−0.024 (4)	0.003 (4)
C1	0.041 (6)	0.055 (8)	0.050 (7)	−0.002 (5)	0.001 (5)	0.017 (5)
C2	0.032 (5)	0.048 (7)	0.046 (6)	0.008 (5)	0.005 (4)	0.005 (5)
C3	0.036 (6)	0.045 (7)	0.036 (6)	0.005 (5)	−0.009 (4)	−0.003 (5)
C4	0.043 (6)	0.041 (6)	0.040 (5)	0.010 (5)	−0.008 (5)	−0.004 (5)
C5	0.046 (6)	0.051 (7)	0.055 (7)	0.018 (6)	−0.003 (5)	−0.006 (5)
C6	0.042 (7)	0.066 (9)	0.060 (8)	0.019 (6)	0.007 (5)	0.007 (6)
C7	0.036 (6)	0.071 (10)	0.063 (8)	0.006 (6)	0.015 (5)	0.010 (7)
C8	0.079 (9)	0.025 (6)	0.087 (9)	−0.003 (6)	0.010 (7)	0.008 (6)
C9	0.086 (9)	0.045 (8)	0.052 (8)	−0.001 (7)	−0.019 (7)	−0.007 (6)
C10	0.084 (9)	0.036 (6)	0.043 (6)	0.015 (6)	−0.005 (6)	−0.003 (5)
C11	0.063 (8)	0.041 (7)	0.024 (6)	0.021 (6)	0.007 (5)	0.010 (5)
C12	0.076 (9)	0.047 (7)	0.045 (6)	0.029 (7)	0.021 (6)	0.011 (6)
C13	0.084 (10)	0.051 (9)	0.056 (8)	0.034 (8)	0.022 (7)	0.011 (6)
C14	0.104 (12)	0.049 (8)	0.057 (8)	0.024 (8)	0.011 (7)	−0.003 (7)
C15	0.105 (12)	0.045 (8)	0.047 (7)	0.019 (7)	−0.005 (7)	−0.011 (6)
C16	0.060 (10)	0.089 (15)	0.128 (17)	0.026 (7)	0.030 (8)	0.031 (13)
C17	0.037 (6)	0.040 (7)	0.075 (10)	−0.011 (5)	−0.017 (6)	0.013 (6)
C18	0.057 (7)	0.023 (6)	0.076 (8)	−0.016 (5)	−0.029 (6)	0.011 (5)

### Geometric parameters (Å, °)

**Table:**

Er1—O3^i^	2.202 (6)	C5—C6	1.433 (19)
Er1—O3	2.202 (6)	C5—H5	0.9300
Er1—O1	2.203 (6)	C6—C7	1.254 (19)
Er1—O1^i^	2.203 (6)	C6—H6	0.9300
Er1—N2	2.595 (8)	C7—H7	0.9300
Er1—N2^i^	2.595 (8)	C8—H8A	0.9600
Er1—N1^i^	2.628 (8)	C8—H8B	0.9600
Er1—N1	2.628 (8)	C8—H8C	0.9600
O1—C3	1.321 (12)	C9—C10	1.54 (2)
O1—H1	0.8200	C9—H9A	0.9700
O2—C4	1.389 (13)	C9—H9B	0.9700
O2—C8	1.419 (14)	C10—C15	1.379 (17)
O3—C11	1.334 (13)	C10—C11	1.394 (19)
O4—C12	1.340 (16)	C11—C12	1.450 (17)
O4—C16	1.446 (16)	C12—C13	1.412 (17)
N1—C17	1.433 (14)	C13—C14	1.35 (2)
N1—C1	1.504 (16)	C13—H13	0.9300
N1—H1C	0.9094	C14—C15	1.36 (2)
N2—C9	1.458 (16)	C14—H14	0.9300
N2—C18	1.495 (15)	C15—H15	0.9300
N2—H2	0.9105	C16—H16A	0.9600
C1—C2	1.479 (15)	C16—H16B	0.9600
C1—H1A	0.9700	C16—H16C	0.9600
C1—H1B	0.9700	C17—C18	1.523 (17)
C2—C3	1.387 (15)	C17—H17A	0.9700
C2—C7	1.426 (15)	C17—H17B	0.9700
C3—C4	1.404 (15)	C18—H18A	0.9700
C4—C5	1.355 (15)	C18—H18B	0.9700
			
O3^i^—Er1—O3	100.6 (4)	C5—C4—C3	121.7 (11)
O3^i^—Er1—O1	89.5 (2)	O2—C4—C3	113.4 (8)
O3—Er1—O1	150.1 (2)	C4—C5—C6	117.5 (12)
O3^i^—Er1—O1^i^	150.1 (2)	C4—C5—H5	121.3
O3—Er1—O1^i^	89.5 (2)	C6—C5—H5	121.3
O1—Er1—O1^i^	95.6 (3)	C7—C6—C5	122.2 (11)
O3^i^—Er1—N2	82.4 (3)	C7—C6—H6	118.9
O3—Er1—N2	71.3 (3)	C5—C6—H6	118.9
O1—Er1—N2	138.4 (3)	C6—C7—C2	121.9 (12)
O1^i^—Er1—N2	74.3 (2)	C6—C7—H7	119.1
O3^i^—Er1—N2^i^	71.3 (3)	C2—C7—H7	119.1
O3—Er1—N2^i^	82.4 (3)	O2—C8—H8A	109.5
O1—Er1—N2^i^	74.3 (2)	O2—C8—H8B	109.5
O1^i^—Er1—N2^i^	138.4 (3)	H8A—C8—H8B	109.5
N2—Er1—N2^i^	138.4 (4)	O2—C8—H8C	109.5
O3^i^—Er1—N1^i^	137.8 (3)	H8A—C8—H8C	109.5
O3—Er1—N1^i^	73.6 (2)	H8B—C8—H8C	109.5
O1—Er1—N1^i^	80.0 (2)	N2—C9—C10	107.3 (10)
O1^i^—Er1—N1^i^	72.0 (2)	N2—C9—H9A	110.3
N2—Er1—N1^i^	130.8 (3)	C10—C9—H9A	110.3
N2^i^—Er1—N1^i^	66.5 (3)	N2—C9—H9B	110.3
O3^i^—Er1—N1	73.6 (2)	C10—C9—H9B	110.3
O3—Er1—N1	137.8 (3)	H9A—C9—H9B	108.5
O1—Er1—N1	72.0 (2)	C15—C10—C11	121.3 (13)
O1^i^—Er1—N1	80.0 (2)	C15—C10—C9	122.4 (12)
N2—Er1—N1	66.5 (3)	C11—C10—C9	116.4 (10)
N2^i^—Er1—N1	130.8 (3)	O3—C11—C10	122.6 (10)
N1^i^—Er1—N1	138.0 (4)	O3—C11—C12	118.9 (11)
C3—O1—Er1	133.1 (6)	C10—C11—C12	118.4 (11)
C3—O1—H1	107.7	O4—C12—C13	127.4 (13)
Er1—O1—H1	107.7	O4—C12—C11	115.7 (10)
C4—O2—C8	116.5 (9)	C13—C12—C11	116.9 (14)
C11—O3—Er1	136.7 (7)	C14—C13—C12	121.5 (14)
C12—O4—C16	115.7 (13)	C14—C13—H13	119.3
C17—N1—C1	115.1 (9)	C12—C13—H13	119.3
C17—N1—Er1	112.2 (6)	C13—C14—C15	121.9 (13)
C1—N1—Er1	114.2 (7)	C13—C14—H14	119.0
C17—N1—H1C	104.5	C15—C14—H14	119.0
C1—N1—H1C	104.4	C14—C15—C10	119.6 (14)
Er1—N1—H1C	105.0	C14—C15—H15	120.2
C9—N2—C18	111.6 (10)	C10—C15—H15	120.2
C9—N2—Er1	115.5 (8)	O4—C16—H16A	109.5
C18—N2—Er1	112.7 (6)	O4—C16—H16B	109.5
C9—N2—H2	105.1	H16A—C16—H16B	109.5
C18—N2—H2	105.1	O4—C16—H16C	109.5
Er1—N2—H2	105.8	H16A—C16—H16C	109.5
C2—C1—N1	110.2 (9)	H16B—C16—H16C	109.5
C2—C1—H1A	109.6	N1—C17—C18	108.9 (9)
N1—C1—H1A	109.6	N1—C17—H17A	109.9
C2—C1—H1B	109.6	C18—C17—H17A	109.9
N1—C1—H1B	109.6	N1—C17—H17B	109.9
H1A—C1—H1B	108.1	C18—C17—H17B	109.9
C3—C2—C7	118.5 (11)	H17A—C17—H17B	108.3
C3—C2—C1	118.1 (10)	N2—C18—C17	108.3 (8)
C7—C2—C1	123.2 (10)	N2—C18—H18A	110.0
O1—C3—C2	120.5 (10)	C17—C18—H18A	110.0
O1—C3—C4	121.3 (9)	N2—C18—H18B	110.0
C2—C3—C4	118.2 (9)	C17—C18—H18B	110.0
C5—C4—O2	124.9 (10)	H18A—C18—H18B	108.4
			
O3^i^—Er1—O1—C3	33.8 (8)	Er1—O1—C3—C2	64.0 (13)
O3—Er1—O1—C3	144.6 (8)	Er1—O1—C3—C4	−116.8 (9)
O1^i^—Er1—O1—C3	−116.7 (9)	C7—C2—C3—O1	178.5 (10)
N2—Er1—O1—C3	−44.0 (10)	C1—C2—C3—O1	−6.0 (15)
N2^i^—Er1—O1—C3	104.5 (9)	C7—C2—C3—C4	−0.7 (15)
N1^i^—Er1—O1—C3	172.7 (9)	C1—C2—C3—C4	174.8 (10)
N1—Er1—O1—C3	−39.1 (8)	C8—O2—C4—C5	−10.6 (15)
O3^i^—Er1—O3—C11	−106.2 (10)	C8—O2—C4—C3	168.9 (10)
O1—Er1—O3—C11	145.9 (9)	O1—C3—C4—C5	−179.0 (10)
O1^i^—Er1—O3—C11	45.5 (10)	C2—C3—C4—C5	0.2 (16)
N2—Er1—O3—C11	−28.1 (9)	O1—C3—C4—O2	1.5 (14)
N2^i^—Er1—O3—C11	−175.4 (10)	C2—C3—C4—O2	−179.3 (9)
N1^i^—Er1—O3—C11	116.9 (10)	O2—C4—C5—C6	179.7 (10)
N1—Er1—O3—C11	−29.0 (11)	C3—C4—C5—C6	0.2 (17)
O3^i^—Er1—N1—C17	107.2 (7)	C4—C5—C6—C7	0(2)
O3—Er1—N1—C17	19.4 (8)	C5—C6—C7—C2	0(2)
O1—Er1—N1—C17	−157.9 (7)	C3—C2—C7—C6	0.9 (18)
O1^i^—Er1—N1—C17	−58.7 (7)	C1—C2—C7—C6	−174.4 (13)
N2—Er1—N1—C17	18.5 (6)	C18—N2—C9—C10	−154.4 (9)
N2^i^—Er1—N1—C17	153.1 (7)	Er1—N2—C9—C10	75.2 (10)
N1^i^—Er1—N1—C17	−107.1 (7)	N2—C9—C10—C15	122.1 (13)
O3^i^—Er1—N1—C1	−119.5 (7)	N2—C9—C10—C11	−58.5 (14)
O3—Er1—N1—C1	152.8 (6)	Er1—O3—C11—C10	52.0 (14)
O1—Er1—N1—C1	−24.5 (7)	Er1—O3—C11—C12	−124.9 (10)
O1^i^—Er1—N1—C1	74.7 (7)	C15—C10—C11—O3	177.0 (10)
N2—Er1—N1—C1	151.9 (7)	C9—C10—C11—O3	−2.4 (16)
N2^i^—Er1—N1—C1	−73.6 (8)	C15—C10—C11—C12	−6.0 (17)
N1^i^—Er1—N1—C1	26.3 (6)	C9—C10—C11—C12	174.5 (9)
O3^i^—Er1—N2—C9	69.5 (9)	C16—O4—C12—C13	−7.5 (19)
O3—Er1—N2—C9	−34.5 (9)	C16—O4—C12—C11	170.7 (12)
O1—Er1—N2—C9	150.1 (8)	O3—C11—C12—O4	0.9 (14)
O1^i^—Er1—N2—C9	−129.4 (9)	C10—C11—C12—O4	−176.1 (11)
N2^i^—Er1—N2—C9	19.2 (8)	O3—C11—C12—C13	179.3 (10)
N1^i^—Er1—N2—C9	−81.1 (9)	C10—C11—C12—C13	2.3 (15)
N1—Er1—N2—C9	144.9 (9)	O4—C12—C13—C14	177.5 (12)
O3^i^—Er1—N2—C18	−60.3 (7)	C11—C12—C13—C14	−0.7 (17)
O3—Er1—N2—C18	−164.3 (7)	C12—C13—C14—C15	3(2)
O1—Er1—N2—C18	20.2 (8)	C13—C14—C15—C10	−6(2)
O1^i^—Er1—N2—C18	100.8 (7)	C11—C10—C15—C14	8(2)
N2^i^—Er1—N2—C18	−110.7 (7)	C9—C10—C15—C14	−172.5 (12)
N1^i^—Er1—N2—C18	149.1 (6)	C1—N1—C17—C18	177.7 (9)
N1—Er1—N2—C18	15.1 (6)	Er1—N1—C17—C18	−49.4 (9)
C17—N1—C1—C2	−159.7 (9)	C9—N2—C18—C17	−177.3 (10)
Er1—N1—C1—C2	68.3 (10)	Er1—N2—C18—C17	−45.5 (9)
N1—C1—C2—C3	−58.1 (13)	N1—C17—C18—N2	63.8 (10)
N1—C1—C2—C7	117.1 (12)		

Symmetry codes: (i) −*x*+2, −*y*+2, *z*.

### Hydrogen-bond geometry (Å, °)

**Table:**

*D*—H···*A*	*D*—H	H···*A*	*D*···*A*	*D*—H···*A*
C16—H16A···O4^ii^	0.96	2.70	3.49 (3)	140
N2—H2···O2^i^	0.91	2.34	3.230 (10)	166
N1—H1C···O4^i^	0.91	2.59	3.462 (12)	162
O1—H1···O2	0.82	2.21	2.646 (9)	113

Symmetry codes: (ii) −*x*+3, −*y*+2, *z*; (i) −*x*+2, −*y*+2, *z*.
